# The Open Perimetry Initiative: A framework for cross-platform development for the new generation of portable perimeters

**DOI:** 10.1167/jov.22.5.1

**Published:** 2022-04-06

**Authors:** Iván Marín-Franch, Andrew Turpin, Paul H. Artes, Luke X Chong, Allison M. McKendrick, Karam A. Alawa, Michael Wall

**Affiliations:** 1 Computational Optometry, Atarfe, Spain; 2 Southwest Eye Institute, Tavistock, UK; 3 School of Computing and Information Systems, University of Melbourne, Melbourne, Victoria, Australia; 4 University of Plymouth, Plymouth, UK; 5 School of Medicine (Optometry), Deakin University, Geelong, Australia; 6 Department of Optometry and Vision Sciences, University of Melbourne, Melbourne, Victoria, Australia; 7 Departments of Neurology and Ophthalmology and Visual Sciences, University of Iowa, College of Medicine, Iowa City, Iowa, USA

## Abstract

The Open Perimetry Initiative was formed in 2010 with the aim of reducing barriers to clinical research with visual fields and perimetry. Our two principal tools are the Open Perimetry Interface (OPI) and the visualFields package with analytical tools. Both are fully open source. The OPI package contains a growing number of drivers for commercially available perimeters, head-mounted devices, and virtual reality headsets. The visualFields package contains tools for the analysis and visualization of visual field data, including methods to compute deviation values and probability maps. We introduce a new frontend, the opiApp, that provides tools for customization for visual field testing and can be used as a frontend to run the OPI. The app can be used on the Octopus 900 (Haag-Streit), the Compass (iCare), the AP 7000 (Kowa), and the IMO (CREWT) perimeters, with permission from the device manufacturers. The app can also be used on Android phones with virtual reality headsets via a new driver interface, the PhoneHMD, implemented on the OPI. The use of the tools provided by the OPI library is showcased with a custom static automated perimetry test for the full visual field (up to 50 degrees nasally and 80 degrees temporally) developed with the OPI driver for the Octopus 900 and using visualFields for statistical analysis. With more than 60 citations in clinical and translational science journals, this initiative has contributed significantly to expand research in perimetry. The continued support of researchers, clinicians, and industry are key in transforming perimetry research into an open science.

## Introduction

The advent of consumer electronics with high-quality display technology (Anthes, García-Hernández, Wiedemann, & Kranzlmüller, 2016) has paved the way for a new generation of portable devices for visual field testing. The transition from traditional projection perimetry to display-based perimetry requires rethinking and adaptation of conventional perimetry methods, and it also provides an opportunity to revise and improve.

The Open Perimetry Initiative is an open-source project that started in 2010 with the goal of alleviating the difficulties of using commercial and experimental ophthalmic devices in vision research. The initiative has evolved beyond its original goal to include features that facilitate the development of new paradigms, standards, and good practices that exploit the technological advantages of portable devices. To maximize accessibility of novel perimetry methods and techniques, implementations using the Open Perimetry libraries should be an open source under the GNU General Public License. To avoid possible legal ramifications, permission from device manufacturers is required before the Open Perimetry Interface (OPI) code is used on their commercial instruments (Turpin, Artes, & McKendrick, 2012).

The key product of the Open Perimetry Initiative is the OPI (Turpin et al., 2012). The OPI not only provides drivers for an increasing number of devices, but it also sets standards and protocols for the implementation of custom visual field tests so that they can be run seamlessly on different instruments, with one implementation for many devices. Furthermore, the OPI can be run in simulation mode, so that new perimetric procedures can be implemented, debugged, and assessed before they are ported to the actual test device. The other key product of the Open Perimetry Initiative is the R (R Core Team, 2022) package visualFields (Marín-Franch & Swanson, 2013), a tool for the statistical analysis and visualization of perimetry results. Until now, the OPI and visualFields solutions have been developed largely independently from one another. However, the recently published shiny package (Chang, Cheng, Allaire, Xie, & McPherson, 2020; https://shiny.rstudio.com) makes it possible to develop cross-platform applications with frontends that integrate the OPI and visualFields software.

The purpose of this paper is to describe the recent advances in the OPI open-source library. A new interface, the PhoneHMD has been included for Android phones with headsets that are compatible with Google Cardboard. The visualFields package has been rewritten to include new interactive features and make it more compatible with the OPI drivers. A frontend app has also been developed that wraps the functionality provided by the OPI. This new frontend, the opiApp, consists of a series of tools to manage databases of study participants, generate custom perimetry grids, and run the same methods on different devices, including the Octopus 900, the AP-7000, and Compass perimeters, and headset devices such as the IMO and the Android phones. The use of these new tools is showcased with examples of published research and ongoing studies.

In addition to the development of novel paradigms, methods, and software, the optical properties of new devices need to be characterized. This includes spatial and grey level resolution along with the effects of chromatic and achromatic aberrations. Further, it is necessary to develop adequate methods for display calibration and for compensating observers’ refractive errors. These considerations are beyond the scope of this paper.

## Methods

The OPI has drivers for the Octopus 900 perimeter (Haag-Streit AG, Köniz, Switzerland), the Compass microperimeter (iCare, Finland), and the AP-7000 perimeter (Kowa, Torrance, CA, USA). New drivers have now been incorporated for the IMO (CREWT Medical Systems, Tokyo, Japan) and Android phones with headsets that are compatible with the Google Cardboard software development kit (Google Inc., Mountain View, CA, USA). Drivers for devices such as the VIVE Pro Eye (HTC Corporation, Taoyuan City, Taiwan) and AVA Advanced Vision Analyzer (Elisar, India) are being developed.

### OPI conventions and standards

The OPI implementations and drivers follow conventions and standards that not only accelerate software development, but also enable the creation of custom tests, perimetric algorithms, and procedures. These can all be used with different computer operating systems, programming languages, and with different commercial and experimental perimeters. The key OPI commands are described more fully elsewhere (Turpin et al., 2012) but are listed here for completeness:

• opiInitialize(): open connection with the perimeter and initialize it,
• opiQueryDevice(): get information about the perimeter,
• opiSetBackground(): set the background color, luminance, fixation point, etc.,
• opiPresent(): Present a stimulus and return observer's response, and
• opiClose(): close the connection with the perimeters.

There are three distinct types of visual field stimuli that can be presented with the opiPresent() command: static, temporal, and kinetic. The static type is used for static automated perimetry (Aulhorn & Harms, 1967; Fankhauser, Koch, & Roulier, 1972; Koch, Roulier, & Fankhauser, 1972). The temporal type is used to generate stimuli that vary over time and that are supported by the underlying hardware, such as a counterphase modulated sine-wave grating of frequency-doubling perimetry (Kelly, 1966; Maddess & Henry, 1992) or the counterphase square-sine flicker stimulus presentation of contrast sensitivity perimetry (Swanson, Malinovsky, Dul, Malik, Torbit, Sutton, & Horner, 2014). The kinetic type can be used to present moving stimuli specified according to the nomenclature introduced by Hans Goldmann (Goldmann, 1999). The level of customization that the OPI offers depends on hardware limitations. For the PhoneHMD OPI, it is possible to define the dynamic range and contrast steps, the stimulus size, color, presentation time, and the response window. It is also possible to set the speed, temporal, and spatial properties of the stimuli.

The opiPresent() command returns the response of the observer (usually whether the response button was pressed or not after a stimulus presentation, along with the time between onset of stimulus presentation and response in milliseconds), and the *x* and *y* pupil position at the time the button was pressed in degrees of visual angle (if hardware allows). The accuracy and precision of all estimates depend on the underlying hardware.

It is possible to interact with the perimeter via the opiPresent() command and build a stimulus-response logic from which complex test procedures can be constructed. Several common procedures are already built into the OPI package:

• MOCS(): The method of constant stimulus or MOCS (Fechner, 1966),
• fourTwo(): The 4-2 dB staircase (Bebie, Fankhauser, & Spahr, 1976; Johnson, Chauhan, & Shapiro, 1992),
• FT(): The full threshold algorithm (Bebie et al., 1976; Johnson et al., 1992), and
• ZEST(): The Bayesian QUEST and ZEST algorithms (King-Smith, Grigsby, Vingrys, Benes, & Supowit, 1994; Turpin, McKendrick, Johnson, & Vingrys, 2003; Watson & Pelli, 1983).

The implementation of the aforementioned OPI commands and drivers are necessarily different for each different perimeter; however, the specific implementation can be selected with the command opiChoose(). Thus, once a specific perimetry driver — Octopus900, KowaAP7000, Compass, IMO, PhoneHMD, or other — has been selected, a dispatcher is set in place so that the same OPI commands listed earlier can be used without change with all supported hardware.

### The visualFields analytical tool

The visualFields package has undergone a major revision moving from version 0.6 (Marín-Franch & Swanson, 2013) to version 1.0.1 introduced here. Its core functionality is the same but the code has been simplified, and a number of conventions have been adopted for clarity and simplicity. An effort has been made to improve its transparency and the reproducibility of its methods. For instance, the SUNY-IU dataset of healthy subjects that was used in the previous version to generate normative values has been incorporated into the package, vfctrSunyiu24d2, along with a function that generates the normative reference values. Thus, the command nvgenerate(vfctrSunyiu24d2) generates pointwise normative values and the command nvgenerate(vfctrSunyiu24d2, method = “smooth”) generates the normative values used in the visualFields 0.6 (Marín-Franch & Swanson, 2013) using the smoothing techniques as those introduced by Heijl and colleagues for the Statpac 2 (Heijl, Lindgren, & Olsson, 1987; Heijl, Lindgren, Lindgren, Olsson, Asman, Myers, & Patella, 1991). Normative datasets, vfctrIowaPC26 and vfctrIowaPeri, and reference values generated with the function nvgenerate for the custom tests used to study the advantages of exploring the full visual field are also included in the package (Marín-Franch, Artes, Chong, Turpin, & Wall, 2018; Wall, Lee, Wanzek, Chong, & Turpin, 2020; Wall, Lee, Wanzek, Zamba, Turpin, Chong, & Marín-Franch, 2019; Wall, Subramani, Chong, Galindo, Turpin, Kardon, Thurtell, Bailey, & Marín-Franch, 2019).

### The opiApp

The R package Shiny (Chang et al., 2020) was used to develop the opiApp, a graphical frontend that interacts with the OPI and can be used to run perimetry on conventional and custom grids of test locations. With the opiApp, it is possible to configure the device and perimetry settings, define the dynamic range of the dB scale and step size, obtain the luminance profile of Android devices (i.e. the correspondence between pixel value and physical luminance), manage patient datasets, and run static automated perimetry using ZEST, MOCS, 4-2 staircase, and Full-Threshold paradigms.

### Software and data availability

The release version of OPI can be found at https://CRAN.R-project.org/package=OPI. The release version of visualFields can be found at https://CRAN.R-project.org/package=visualFields. The development version of OPI can be found at https://github.com/turpinandrew/OPI. The development version of visualFields can be found at https://github.com/imarinfr/vf1. The driver to run perimetry on Android phones with headsets that are compatible with Google Cardboard can be found at https://github.com/imarinfr/opiPhoneHMD. The opiApp frontend for the OPI can be found at https://github.com/imarinfr/opiApp. Most datasets used in this paper can be found within the visualFields package. The dataset of healthy subjects for the full visual field can be found in the visualFields package as well as in https://www.sciencedirect.com/science/article/pii/S2352340918311570. The applications developed based on the OPI and visualFields packages and presented in the paper, as well as the datasets are available from the corresponding author on reasonable request.

## Results

**Figure 1.**
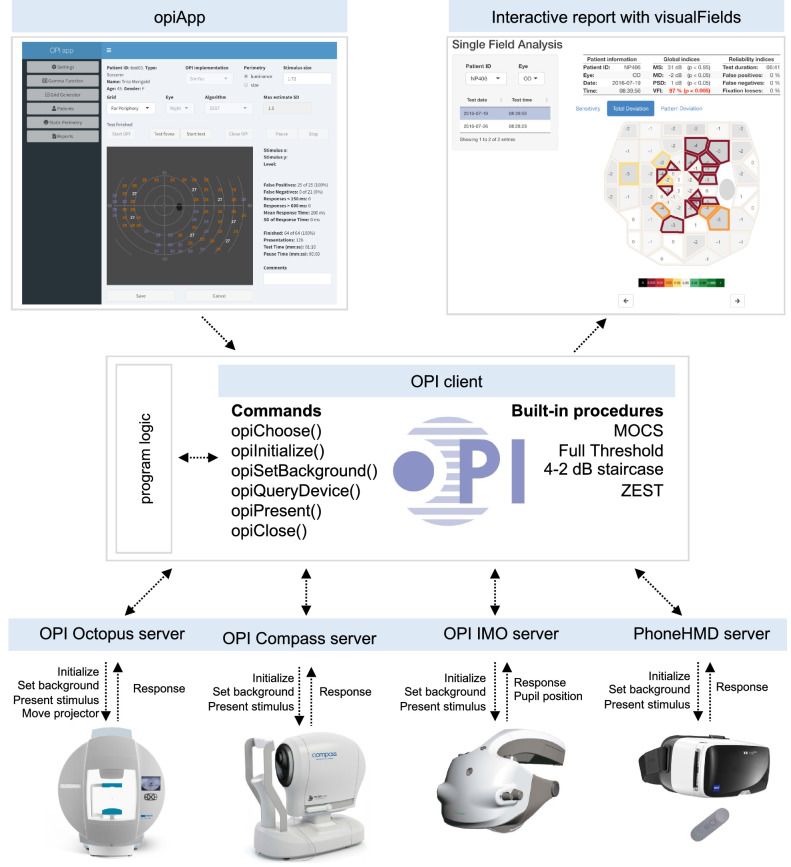
**Illustration of the OPI architecture.** Top left is a graphical interface generated in shiny for a program logic that can be run on any device at the bottom through the OPI client (center) as it dispatches commands via the OPI servers. Once a suitable dataset of healthy controls has been collected, statistical analyses as the one in the top right can be generated with the visualFields package.

Figure 1 illustrates the OPI architecture. Once the hardware is selected, the R OPI client dispatches the commands to connect, disconnect, set background and other settings, and present stimuli to the corresponding OPI server (Octopus 900, AP-7000, Compass, IMO, PhoneHMD, etc.), which ultimately communicates the commands to the hardware. The server then waits for the hardware to send its state (machine initialized, background lit, pupil position, clicker pressed within a time response window after stimulus onset, etc.) and communicates the response to the OPI client. A frontend, as the opiApp shown in the top left of Figure 1, can be developed on top of a program logic to run conventional or custom visual field tests on both regular or irregular grids of test locations with the OPI built-in algorithms, such as Zippy Estimation of Thresholds (ZEST). It also has the capability to run other procedures, including a suprathreshold test due to Aulhorn (Aulhorn & Harms, 1967) or the binocular Esterman test (Esterman, 1982). The frontend and the backend are running in parallel separated processes. The frontend communicates actions for the backend to process and the backend interfaces with the perimetry devices via the OPI. This way, the opiApp is replaceable by any other frontend developed in R, Java, Python, or any other suitable programming language.

**Figure 2.**
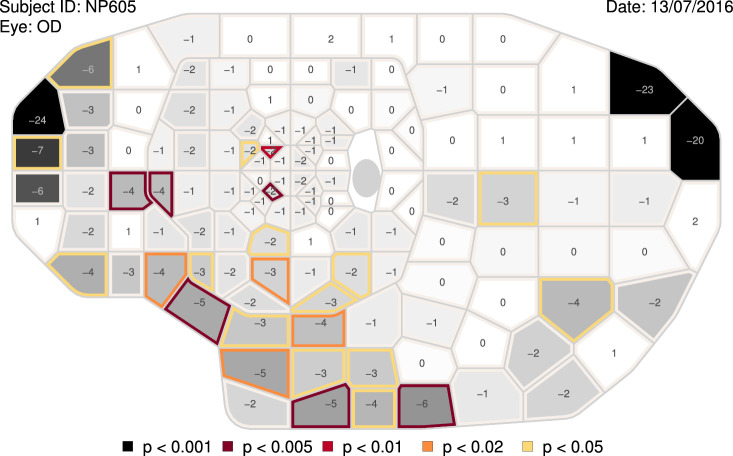
**Combined grayscale sensitivity and color-coded total-deviation map.** The representation of the full visual field results are composites of two tests taken on the same day: one spanning the central 26 degrees of the visual field and another from 26 degrees to 50 degrees nasally, to 80 degrees temporally, to 46 degrees superiorly, and to 50 degrees inferiorly. The values shown at each location are total deviations, departures in sensitivity from the mean normal sensitivities for age-matched controls. The background grayscale of each tile represents the estimated sensitivity at the corresponding visual field location, where darker means lower sensitivity. Tiles whose border is shown in color are significantly depressed according to the statistical analysis of the total-deviation map. The tiles involving each visual field location were obtained using Voronoi tessellation (Aurenhammer & Klein, 1999; Kucur, Holló, & Sznitman, 2018) to achieve an efficient representation for both highly irregular grids. Voronoi tessellations are a partitioning of a surface into regions so that the center of each cell is its mean (center of mass). Every point in a given Voronoi polygon is closer to its generating point than to any other cell.

The OPI was used to run a series of tests of the full visual field (Marín-Franch et al., 2018; Wall et al., 2020; Wall, Lee, et al., 2019; Wall, Subramani, et al., 2019). A publicly available dataset of 98 eyes of 98 healthy subjects (Marín-Franch et al., 2018) was used to derive normative values with the visualFields R package. Figure 2 shows a statistical analysis of the results for a specific full visual field, which consists of the combined analysis of two tests taken on the same day, one for the central visual field and another for the far periphery (Marín-Franch et al., 2018; Wall et al., 2020; Wall, Lee, et al., 2019; Wall, Subramani, et al., 2019). A dataset of healthy eyes (Marín-Franch et al., 2018) is incorporated in the visualFields version 1.0.1 for the central and peripheral tests, vfctrIowaPC26 and vfctrIowaPeri. A script that generates figures for all subjects in those datasets is provided as supplemental material with the name vfPlotFullField.r.

**Figure 3.**
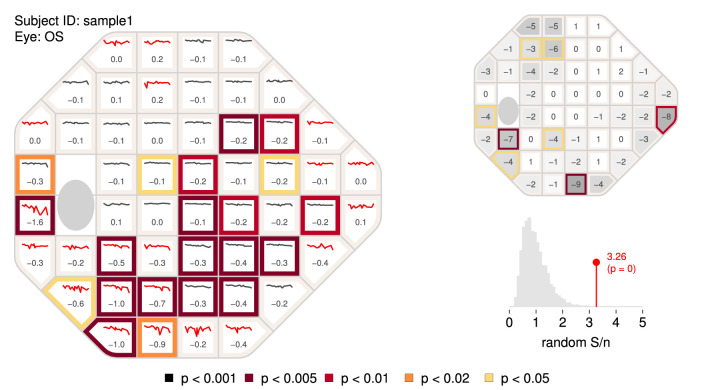
**The PoPLR analysis.** The left panel shows the slopes at each visual field location obtained with pointwise linear regression of total deviation values over time along with sparklines representing the values over the whole series. The colors at the border of the tiles categorize the *p* values of the one-tail *t*-test with the alternative that the slope is negative. To identify highly variable series of visual fields, the sparklines are shown in red if the median absolute deviation of the residuals from linear regression were greater than 2 dB. The top right graph is the combined grayscale sensitivity and total-deviation map of the baseline sensitivity values (intercept of pointwise regression on sensitivities). The bottom right function shows the histogram of random *S* / *n*, where *n* is the number (52 in this case) of regression analyses performed obtained by permuting the series as part the of PoPLR analysis. The *p* value for the PoPLR analysis testing whether there is deterioration is shown next to the value of the observed *S* / *n* statistic.

The visualFields package also offers tools to analyze longitudinal data, including pointwise linear regression, and the permutation of pointwise linear regression, or PoPLR (Marín-Franch, Artes, Turpin, & Racette, 2021; O'Leary, Chauhan, & Artes, 2012). Figure 3 shows a brief report (generated with the script vfPoPLRAnalysis.r provided as supplemental material) and with the vfpwgSunyiu24d2 dataset (Artes, O'Leary, Nicolela, Chauhan, & Crabb 2014), which was collected with the Humphrey Field Analyzer (Carl Zeiss Meditec, Inc., Dublin, CA, USA). The normative values to obtain total deviation values and probability maps were generated using the dataset, vfctrSunyiu24d2, from a prospective longitudinal study conducted at Indiana University and State University of New York, SUNY (Marín-Franch & Swanson, 2013). The normative values were obtained with the command nvgenerate(vfctrSunyiu24d2).

The ZEST algorithm used to measure the visual field (Marín-Franch et al., 2018; Wall et al., 2020; Wall, Lee, et al., 2019; Wall, Subramani, et al., 2019) in Figure 2 can be invoked by the opiApp. This specific implementation has a bimodal prior probability mass function with one peak centered at 0 dB to model sensitivities of damaged locations and another peak that depends on sensitivity estimates in neighboring locations (Vingrys & Pianta, 1998) applied using a growth algorithm that defines the order at which locations at different regions of the visual field are tested. Supplementary Figure S1 illustrates the growth algorithm for the 24-2 grid of test locations. A conventional 24-2 grid was used instead of the irregular one in Figure 2 for clarity of illustration. Supplementary Figure S2 shows the growth-algorithm setup for custom central and far periphery irregular grids of test locations.

**Figure 4.**
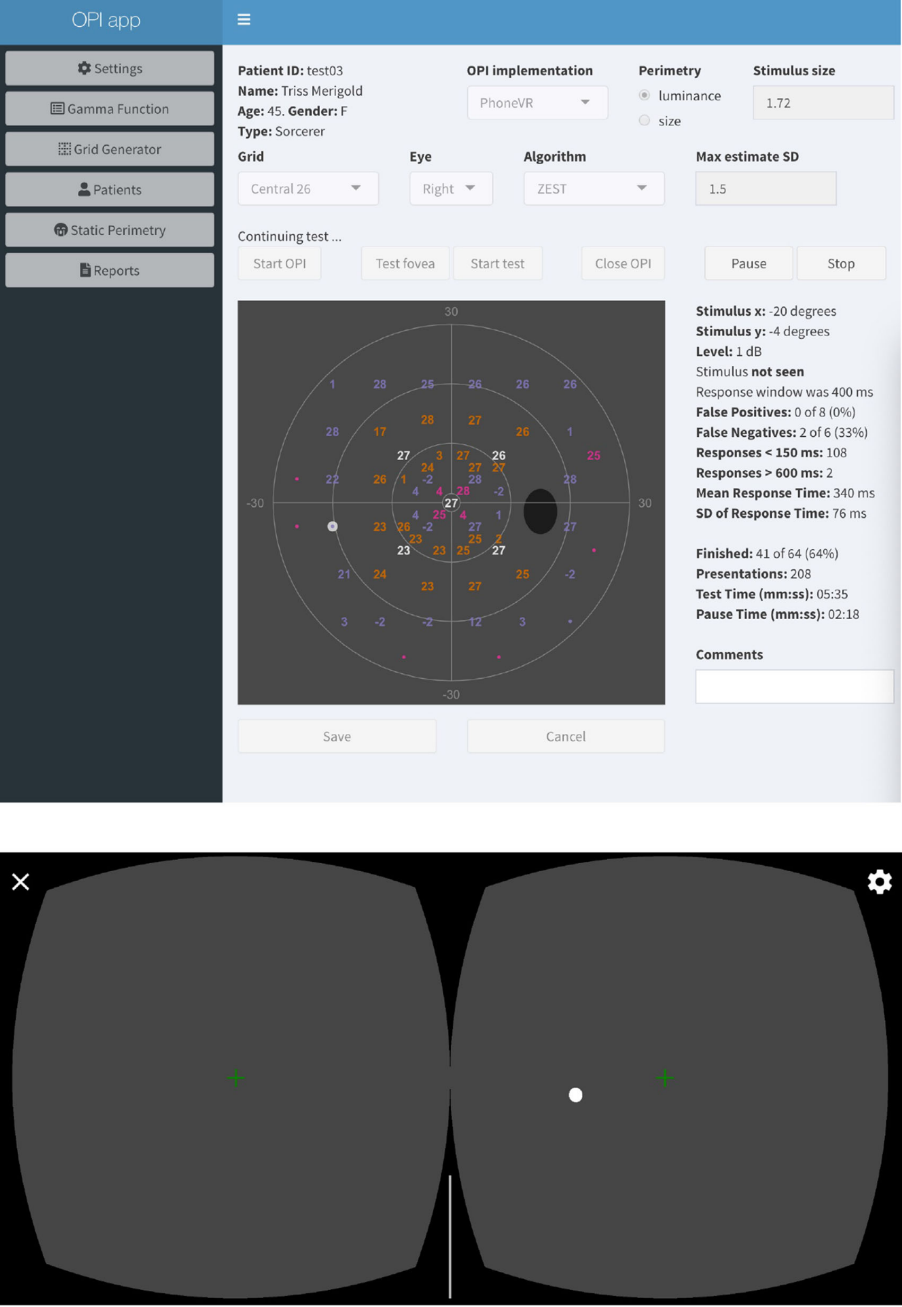
**The** **opiApp on the PhoneHMD OPI.** The ZEST algorithm for luminance (white-on-white) perimetry for a custom irregular grid of test locations is executed for a (fictitious) patient. The opiApp (top) sends commands to an Android Samsung Galaxy S9 phone (bottom) to generate white visual stimuli at different intensities and at different distances from the fixation point (green cross). At each presentation, the web app updates and shows the interim results of the test.

Figure 4 shows a snapshot of the ZEST algorithm with a growth pattern algorithm to an irregular grid of test locations that corresponds to the central part in the full visual field tests (Marín-Franch et al., 2018; Wall et al., 2020; Wall, Lee, et al., 2019; Wall, Subramani, et al., 2019) with the opiApp running with the PhoneHMD OPI. The opiPhone server permits to present stimuli in either or both eyes. Likewise, the background and fixation targets can be set for the either or both eyes. The gamma function was obtained with the option *Gamma Function* provided by the opiApp frontend (see Supplementary Figure S3). Other irregular grids can be designed with the option *Grid Generator* (see Supplementary Figure S4).

## Discussion

Visual fields are among the most often conducted vision tests, second only after visual acuity. Thousands of tests are conducted every day. Although there have been many interesting advances in perimetry (Greenfield, Deiner, Nguyen, Wollstein, Damato, Backus, Wu, Schuman, & Ou, 2022; Jones, 2020; Prager, Kang, & Tanna, 2021; Prince, Thompson, Mwanza, Tolleson-Rinehart, & Budenz, 2021; Stapelfeldt, Kucur, Huber, Höhn, & Sznitman, 2021; Turpin, Myers, & McKendrick, 2016), the community pursuing clinical visual field research remains relatively small. To many newcomers, perimetry first appears as something of a black art, with a specialist terminology somewhat at odds with mainstream psychophysics.

In addition, whereas researchers have always shared resources, such as data and analysis routines with each other, this has traditionally been in an informal way, more open to those who are already part of an established research community than newcomers. More formal arrangements often come with bureaucratic and legal burdens. It has only been relatively recent that clinicians and scientists have begun to share datasets (Bryan, Vermeer, Eilers, Lemij, & Lesaffre, 2013; Marín-Franch et al., 2018; Montesano, Chen, Lu, Lee, & Lee, 2022; Swanson, Dul, Horner, & Malinovsky, 2016) and source code openly under the General Public License, such as the GNU GLP (Free Software Foundation, 2022) or the MIT license (Open Source Initiative, 2022). The founding aims of the Open Perimetry Initiative (Turpin et al., 2012) were to foster an unfettered exchange of ideas, tools, and data related to visual fields and perimetry, and thereby to reduce the inefficiencies and inconveniences traditionally associated with research in perimetry.

Perimeters built on consumer hardware could make a real impact (Anthes et al., 2016) on several hitherto unsolved problems in clinical perimetry. For example, if patients could test themselves at home, visual field tests could potentially be performed much more frequently than with current office-based equipment. In turn, frequent testing could lead to a breakthrough in our ability to measure change over time. It is not hard to think of other examples where wider access to visual field tests could improve patient care or increase access to visual assessment in occupational or other relevant applied settings. Furthermore, reducing reliance on expensive perimetry specific hardware may accelerate the input from a new generation of vision scientists to the field of perimetry research. However, to make best use of consumer electronics hardware originally designed for other applications, visual field tests will need to be adapted, along with the statistical analyses and management of the resulting data.

**Figure 5.**
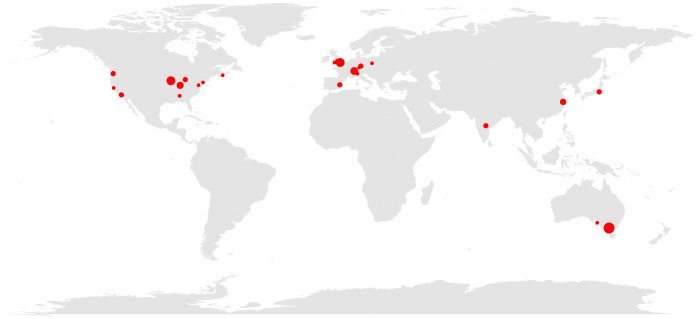
**Geographical map of citations to the OPI and the visualFields R package as listed in Scopus.** The red solid circles demarcate the cities of the first authors’ affiliations. The sizes of the circles represent the number of citations from each city, up to three. As of February 2022, OPI and visualFields related publications received 66 peer-reviewed citations from 12 countries in 4 continents.

By adhering to the open science principles — open source, open data, and open access — this work can be performed transparently and made as widely accessible as possible. Modern visual field research has many worthwhile challenges to be addressed, and we hope that the OPI will act as a catalyst to bring new minds into this domain, to facilitate collaboration between research groups across the globe (Figure 5), and between basic and clinical scientists and industry partners.

## Supplementary Material

Supplement 1

Supplement 2

Supplement 3

Supplement 4

Supplement 5
