# Supplementary material for: The Open Perimetry Initiative: A framework for cross-platform development for the new generation of portable perimeters
Source: J Vis. 2022 Apr 6;22(5):1. doi: 10.1167/jov.22.5.1 (PMC8994165; doi:10.1167/jov.22.5.1)
Supplement: Supplement 1 [file jovi-22-5-1_s001.pdf]

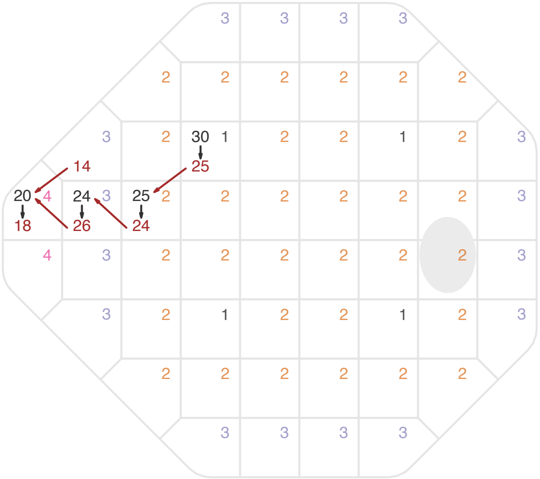

Figure S1. Growth algorithm settings for conventional 24-2 testing. The numerals represent the wave number of the 54 locations of the 24-2 regular grid of test locations for the growth algorithm. Locations corresponding to the primary wave are labeled with the number 1, and locations corresponding to the second, third, and fourth waves are respectively labeled with the numbers 2, 3, and 4. At the beginning, only the four primary locations are tested at random, with the second peak of the ZEST bimodal probability mass function at 30 dB for the Octopus 900. Once a primary location is complete, all neighboring locations that correspond to the second wave open up for testing, with the second peak centered at the sensitivity estimated at its predecessor as illustrated by the red arrows. The black arrows represent the test run for this location, whereas the dark red arrows represent the propagation of sensitivity estimates among neighbor locations.
