# Supplementary material for: The Open Perimetry Initiative: A framework for cross-platform development for the new generation of portable perimeters
Source: J Vis. 2022 Apr 6;22(5):1. doi: 10.1167/jov.22.5.1 (PMC8994165; doi:10.1167/jov.22.5.1)
Supplement: Supplement 2 [file jovi-22-5-1_s002.pdf]

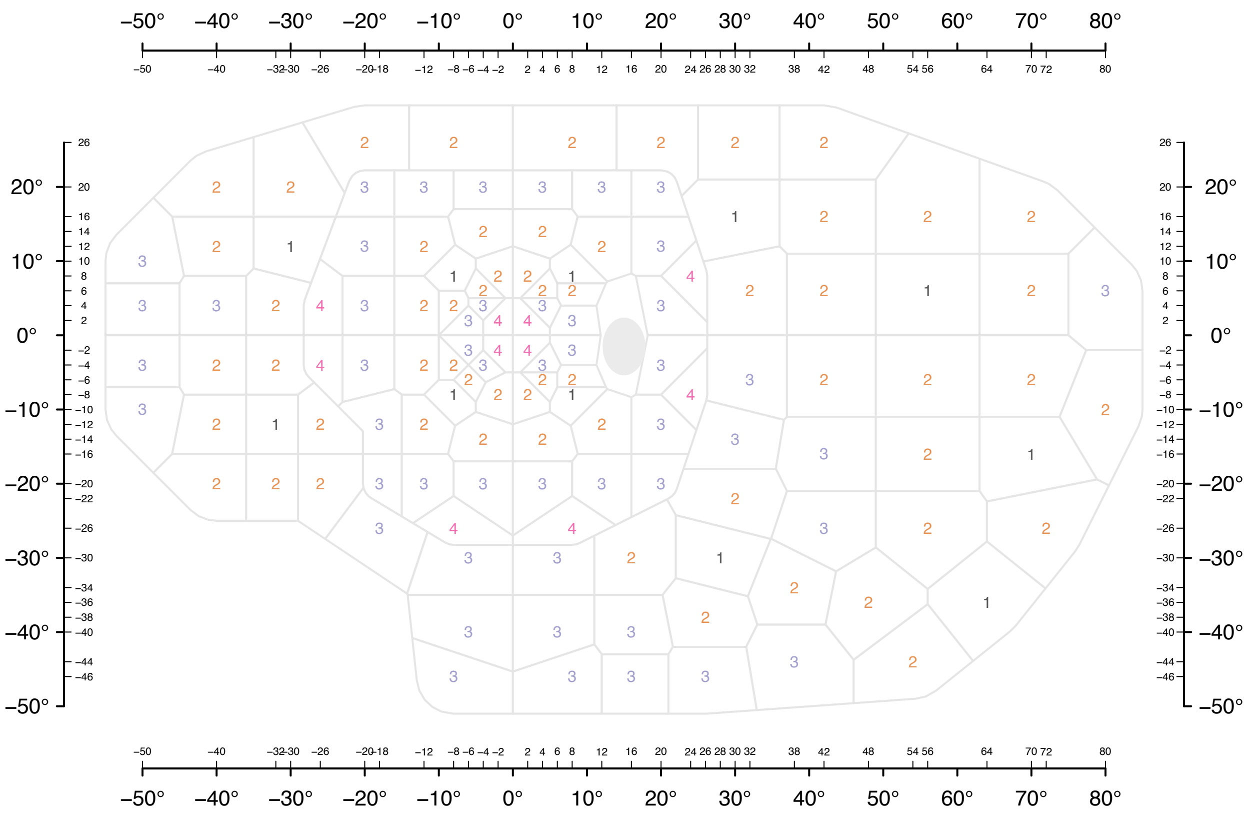

Figure S2. Growth algorithm settings for custom central and far periphery testing. The central grid goes from 26° nasally to 24° temporally and from 20° superiorly to 26° inferiorly and it is superimposed to the far periphery grid. The far periphery grid goes from 50° to 26° nasally, from 24° to 80° temporally, from 26° to 20° superiorly and from26° to 46° inferiorly. Other details are as for **Figure S1**.
