# Supplementary material for: The Open Perimetry Initiative: A framework for cross-platform development for the new generation of portable perimeters
Source: J Vis. 2022 Apr 6;22(5):1. doi: 10.1167/jov.22.5.1 (PMC8994165; doi:10.1167/jov.22.5.1)
Supplement: Supplement 3 [file jovi-22-5-1_s003.pdf]

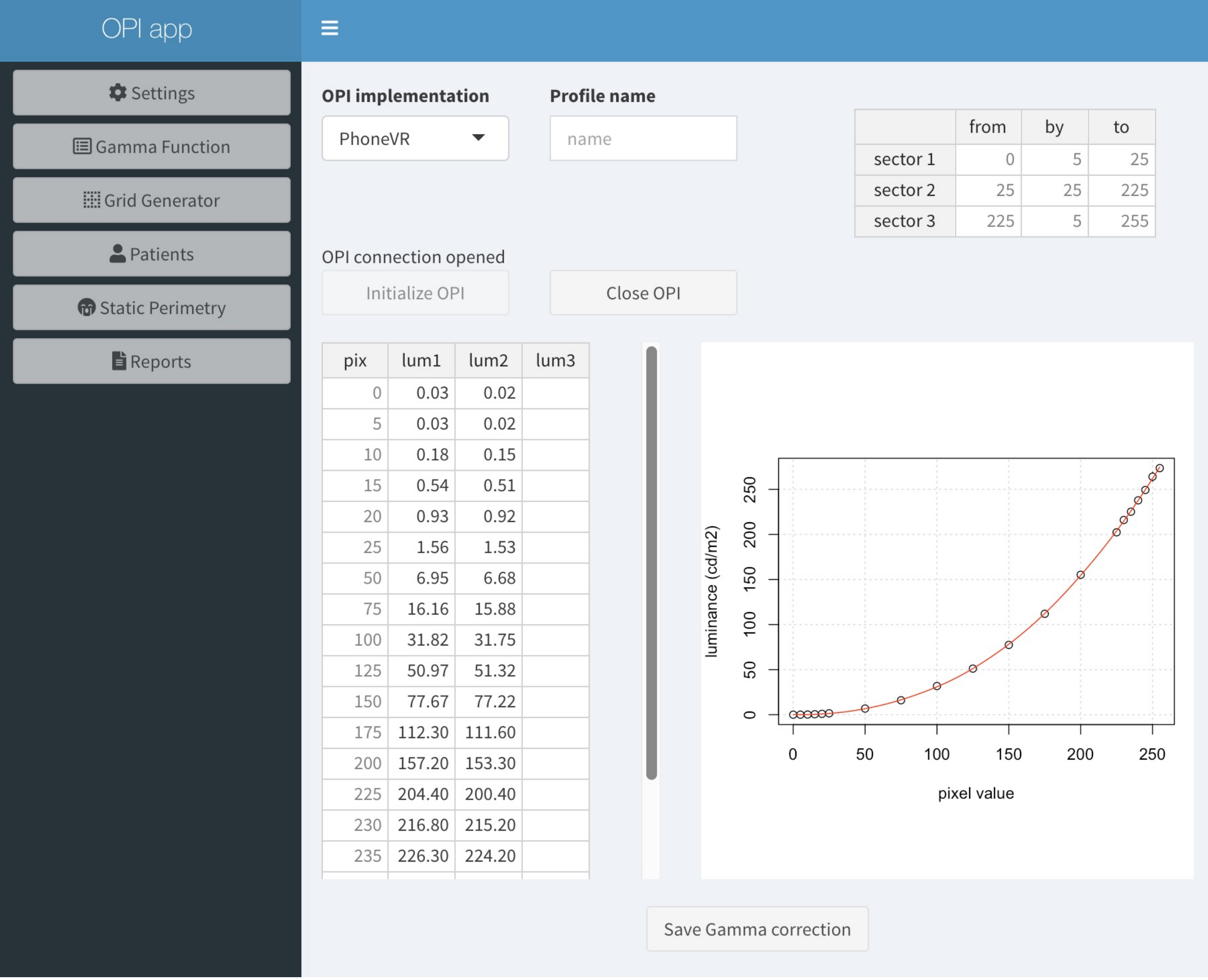

Figure S3. Gamma correction for the Samsung Galaxy S9. Since gamma corrections normally follow a sigmoid function, the measurement points (from 0 to 255 luminance levels) are defined at different resolutions in 3 different sectors (table at the top right). These values are input by the user so that the measurements can be adapted to each phone's display. The app allows up to 3 repetitions at each level (table at the bottom left) to test for device variability. A loess function is fitted to interpolate between measured points. The save button at the bottom records the measurements and a look-up table with the luminance values at each of the 256 levels as fitted by loess. In this example, it appears that the luminance for the Samsung Galaxy S9 is capped as the gamma function did not saturate at the largest luminance levels.
