# Supplementary material for: The Open Perimetry Initiative: A framework for cross-platform development for the new generation of portable perimeters
Source: J Vis. 2022 Apr 6;22(5):1. doi: 10.1167/jov.22.5.1 (PMC8994165; doi:10.1167/jov.22.5.1)
Supplement: Supplement 4 [file jovi-22-5-1_s004.pdf]

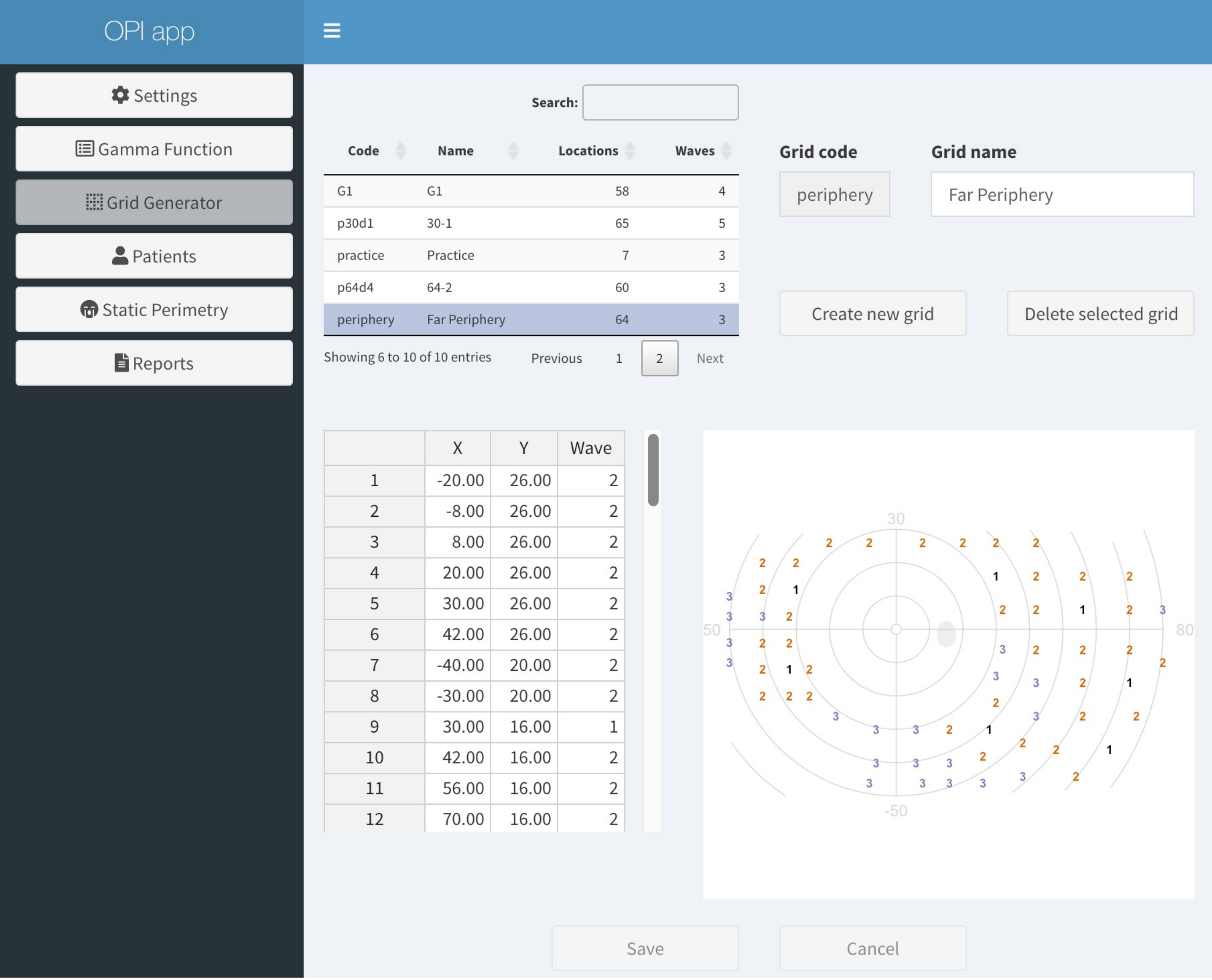

Figure S4. Custom grid generator. Custom grids can also be added for testing with the static automated perimetry algorithms in-built in the OPI. The Wave column in the table at the left are used to define the growth algorithm. The cardinal points belong to the first wave. The sensitivity estimates obtained once they have finished testing propagate to the neighbor locations of the second wave and those to the locations of the third wave, etc. The graph on the right shows the test locations in the grid and the order they are open for testing for the far periphery grid.
